# Consultations

**Published:** 1881-02-20

**Authors:** 


### *CONSULTATIONS.*

We doubt not that all experienced practitioners have learned to be cautious in regard to consultations. It is a lamentable fact that comparatively few physicians are familiar with the code of ethics on this point; and unfortunately there are those who, though acquainted with the code and its principles, yet do not regard them, or who, while observing the letter, manage to violate the spirit of the instrument. These remarks are designed to invite attention to this subject by members of the profession everywhere, so that those who have not read the ethics may perhaps be induced to do so, and that those who have read it may refresh their minds by reading it again. We do not attempt here to detail the particulars of the code, but, reduced to its essence, it simply means this—"Do as you would be done by."

An intelligent gentleman of the profession recently remarked in our hearing—"I will never call another consultation if I can possibly avoid it." When asked the reason why, he stated that the party consulted nine times out of ten done or said something that lost the attending physician the confidence of the sick man's family, and his practice thereafter, or otherwise injured his professional reputation."

We have had the same to occur in our experience. It often occurs that a physician calls in a professional brother, not so much for counsel as for support in cases where he sees increasing alarm and anxiety on the part of the friends. For the physician consulted under such circumstances to suggest an immediate and radical change of treatment, thereby giving the impression that something else was indispensable and imperative, is calculated to injure the physician in attendance. He should rather seek to sustain the family physician, complimenting, if possible, his medical skill, carefully avoiding, either by remark, inuendo, shake of the head, or anyway whatever, doing anything to the prejudice of the attending physician. There is one method of hurting a professional brother which is often done thoughtlessly, but more frequently with intent to injure the physician in charge. It is the relating of cases *just like it* that you have cured. In fact there are a thousand ways of injuring a professional brother without violating the letter of the code. We expect to recur to this subject at a future time.

